# Impact of COVID-19 on physical activity: A rapid review

**DOI:** 10.7189/jogh.12.05003

**Published:** 2022-04-30

**Authors:** Amaryllis H Park, Sinan Zhong, Haoyue Yang, Jiwoon Jeong, Chanam Lee

**Affiliations:** Department of Landscape Architecture & Urban Planning, College of Architecture, Texas A&M University, Texas, USA

## Abstract

**Background:**

Physical activity is a commonly prescribed medicine for people with conditions such as obesity and diabetes who are also at increased risk of being hospitalized or severely ill from COVID-19. However, many people are reporting challenges in engaging in a healthy dose of physical activity amid the pandemic.

**Objective:**

This rapid review synthesizes the current empirical evidence about the impacts of COVID-19 on people’s outdoor physical activity and sedentary behavior while highlighting the role of community environments in promoting or hindering physical activity during the pandemic.

**Methods:**

Literature searches were conducted using keywords related to COVID-19: physical activity, mobility, and lifestyle behaviors. Eligibility criteria were peer-reviewed empirical and quantitative studies published in English, addressing COVID-19 and using physical activity and/or sedentary behavior as the study outcomes.

**Results:**

Out of 61 eligible studies, the majority (78.3%) were conducted in Asian and European countries, with only four (6.7%) being US studies. The results showed that COVID-19 was linked with significant decreases in mobility, walking, and physical activity, and increases in sedentary activity. A few studies also reported contradicting results including increased uses of parks/trails and increased recreational activity among certain groups of population.

**Conclusions:**

Evidence suggests an overall negative impact of COVID-19 on physical activity, with differential effects across different sub-populations. Significant knowledge gaps are also found in the roles of social and physical attributes that can promote physical activity during pandemics with reduced safety risks.

The spread of coronavirus disease 2019 (COVID-19), classified as a pandemic by the World Health Organization on March 11, 2020, has greatly impacted people’s daily lives globally [1]. The COVID-19 Community Mobility Reports from Google indicate that compared to the pre-COVID-19 baseline (the median value of the five weeks from January 3, 2020 to February 6, 2020), mobility trends during COVID-19 (as of March 9, 2021) showed decreases in most locations including retail and recreation (-14%), transit stations (-34%), grocery stores and pharmacies (-5%), and workplaces (-31%); however, increases have been witnessed in parks (+9%) and residential places (+9%) [2]. This suggests that while people spent more time at home, they also visited parks and open spaces (eg, national parks, public gardens, dog parks) more frequently during the pandemic [2].

Outdoor physical activity holds strong potential as an effective coping and preventive strategy given its many well-documented physical, social, and mental health benefits for people of all ages, especially those with or at risk of developing chronic diseases [3,4]. Many empirical studies suggest that physical activity can prevent chronic diseases (eg, cardiovascular disease, diabetes, and obesity), improve brain health and conditions, promote mental health (eg, reduced depression and anxiety), and reduce falls or fall-related injuries [5,6]. For those with existing health conditions such as diabetes and obesity, physical activity is even more critical as a remedy to treat and manage the conditions [7]. Recognizing these health benefits, many efforts have been initiated by organizations like America Walks and World Health Organization to promote physical activity, which increasingly recognize the importance of environmental approaches. The Surgeon General’s Call to Action to Promote Walking and Walkable Communities released in 2015 by the US Department of Health and Human Services recognizes the importance of active lifestyles and creating walkable environments to support active living [8]. A large body of physical activity literature recognizes the significant roles of community environments (eg, streetscape, esthetics, roads and traffic, neighborhood parks, and activity zones) in promoting physical activity and reducing sedentary behavior [9,10].

While considering health-promotion strategies and interventions, Stokols (1992) emphasized the nature of individuals’ interactions with physical and sociocultural environments from an ecological perspective, focusing on the need of addressing environmental factors to change/promote health-related behaviors [11]. Ecological models have been popularly used as the foundation to understand and develop multilevel approaches of promoting health behaviors. Bronfenbrenner (1994) introduced the ecological systems model, which focused on the impact of the environment on individual development. He saw individual development influenced by multi-layered exposures to various environmental factors over time, categorized into five systems, including microsystem, mesosystem, exosystem, macrosystem, and chronosystem [12]. The quality of each layer can either enhance or restrict individuals’ health-related behaviors.

COVID-19 has brought a new layer of challenges to our health and healthy behaviors. People worldwide are reporting challenges in engaging in a healthy dose of physical activity amid the pandemic, with up to a 50% decrease of physical activity in some areas. This decline is in part due to the limited and inequitable availability of safe outdoor community resources (eg, parks, trails, sports facilities, sidewalks) that support physical activity [13]. Despite the growing recognition of the negative impacts of COVID-19 on people’s health, only a small amount of literature has explored the roles of environmental factors such as neighborhood infrastructure and recreational resources that can help promote or hinder physical activity. Recent reviews showed that the personal, behavioral, and social changes during the pandemic have led to mental distress and illness [14-16]. Reviews of work on children and older adults emphasized the environmental associations of anxiety, depression, and other psychological outcomes (eg, worry, grief) during the COVID-19 pandemic [14,16]. Usher et al. (2020) also conducted a rapid review highlighting the psychological distress (eg, fear, stress, anger, and frustration) associated with behavioral changes (isolation and quarantine) and restricted social interactions (social distancing) [15]. Facing the new normal of everyday life, Megahed & Ghoneim (2020) suggested research areas and questions related to five scopes of post-coronavirus architecture and urbanism (post-pandemic urbanism, public spaces, housing, office spaces, and building and construction technology) that researchers need to address to prevent the virus from spreading [17].

This rapid review aims to show whether COVID-19 has impacted people’s outdoor physical activity and sedentary behavior during the early phases of the pandemic, by synthesizing the current empirical evidence on this topic, and highlighting the roles of community environments in promoting or hindering physical activity during the pandemic. Going beyond the scope of previous literature reviews on COVID-19, this study includes multiple and specific physical activity outcomes (eg, walking and biking), and summarizes several environmental factors shown to be important for promoting physical activity amid the pandemic. By providing a comprehensive and critical examination of the current body of literature on this topic, we aim to provide empirical guidance for policymakers, researchers, and practitioners. This guidance can support those in various professions toward investigating the full range of environmental facilitators and barriers to promoting active living, as an effective way to cope with emotional or mental distress during pandemics like COVID-19.

## METHODS

### Eligibility criteria

We selected studies that met the following four criteria: 1) addressing the impact of COVID-19, 2) including physical activity (eg, step counts, weekly minutes of physical activity, time spent outdoors) and/or sedentary behavior (eg, screen/sitting time, total sedentary time) as study outcomes, 3) peer-reviewed empirical and quantitative studies including both cross-sectional and longitudinal studies, and 4) studies written in English. We excluded studies on health care facilities and professional athletes, given their unique and narrow focuses that are different from our main study aim targeting general populations in their everyday residential communities.

### Search strategy

Literature searches were conducted in PubMed on October 23, 2020, using pre-developed keywords related to COVID-19, physical activity, mobility, and lifestyle behaviors. COVID-19 related search terms included “coronavirus” or “COVID-19” or “coronavirus disease 2019” or “SARS-COV-2” or “Severe Acute Respiratory Syndrome Coronavirus 2”. Physical activity related search terms encompassed “physical activity” or “walk” or “bike” or “sedentary” or “exercise” or “active living” or “transport” or “mobility” (**Table 1**). The PubMed search limiter included articles being published between 2019 and 2020. Additional searches were conducted in Google Scholar on November 1, 2020, to validate and supplement the PubMed search results.

**Table 1:** PubMed search string

Domains	Search string
COVID-19	((wuhan[tw] AND (coronavirus[tw] OR corona virus[tw])) OR coronavirus*[ti] OR COVID*[tw] OR nCov[tw] OR 2019 ncov[tw] OR novel coronavirus[tw] OR novel corona virus[tw] OR covid-19[tw] OR SARS-COV-2[tw] OR Severe Acute Respiratory Syndrome Coronavirus 2[tw] OR coronavirus disease 2019[tw] OR corona virus disease 2019[tw] OR new coronavirus[tw] OR new corona virus[tw] OR new coronaviruses[all] OR novel coronaviruses[all] OR “Severe Acute Respiratory Syndrome Coronavirus 2”[nm] OR 2019 ncov[tw] OR nCov 2019[tw] OR SARS Coronavirus 2[all]) AND (2019[dp]:2020[dp])))
Active living	(Physical activit*) or walk* or sedentary or (moderate activit*) or (vigorous activit*) or exercise or bike or biking or bicycle or bicycling or cycling or recreation* or (active living) or running or jogging or strolling or (screen time) or (sedentary behavior) or play or transport* or transit or bus or (light rail) or travel or trip or mobility or (outdoor time) or leisure*

### Selection process

Eligible studies were selected in two phases using Covidence (www.covidence.org), which is a web-based systematic review software for screening and data extraction. First, studies searched in PubMed were screened based on the titles and abstracts. Second, articles selected from the title and abstract screening went through the full-text sorting. Two researchers completed the screening process independently, and all disagreements were resolved through consensus discussions with a third researcher.

### Quality assessment

Two researchers independently conducted the quality assessment of each study selected from the title and abstract screening and the full-text sorting, using a pre-developed quality assessment tool adapted from established tools for quantitative studies [18,19]. The methodological quality was assessed on a rating scale of zero to six based on the following six criteria: 1) study design (cross-sectional vs longitudinal studies), 2) sample representativeness, 3) large sample size, 4) measurement tool for outcome variables, 5) confounders, and 6) statistical methods. The studies were classified as low-quality, middle-quality, and high-quality based on the rating scores of 0 to 2, 3 to 4, and 5 to 6, respectively.

### Data extraction

Using a pre-established data extraction template, we synthesized the study characteristics related to the fields of publication, study designs, study locations and settings, study participants, confounding/control variables, environmental factors, physical activity outcomes, statistical study findings, and quality assessment results (Table S1 in the **Online Supplementary Document**). Two researchers extracted the data from the selected studies and cross-checked the extracted data. Two other researchers further reviewed the quality of the extracted data.

## RESULTS

### Study identification

**Figure 1:**
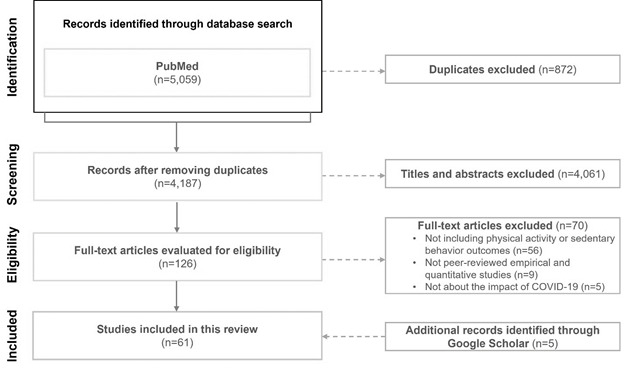
PRISMA flow chart.

**Figure 1** displays the search and selection process that follows the Preferred Reporting Items for Systematic Reviews and Meta-Analyses (PRISMA) guidelines. A total of 5059 records were identified from the PubMed database search. A total of 872 duplicates and 4061 irrelevant records were removed after the title and abstract screening. The remaining 126 articles were further screened based on the full text, which identified 70 ineligible articles. In addition, 5 studies were identified through Google Scholar. This led to the final total of 61 eligible studies for the data extraction and synthesis.

### Characteristics of the included studies

Of the 61 reviewed studies summarized in **Table 2**, the majority were cross-sectional studies (n = 52, 85.2%). Popular fields of publication were health-related (n = 54, 88.5%) including public/global health, medical, and nutrition/physical activity. Most of the studies were conducted in Europe (n = 27, 44.3%), North America (n = 13, 21.3%), or Asia (n = 13, 21.3%). The sample sizes of the reviewed studies ranged between 24 and 7420 000, and over two thirds had a sample of at least 400. The study population covered children and/or adolescents (n = 8, 13.1%), adults (n = 43, 70.5%), and older adults (n = 2, 3.3%). Five domains of physical activity-related outcomes were measured in the reviewed studies: walking and biking (n = 13, 8.1%), physical activity (n = 92, 57.5%), mobility (n = 17, 10.6%), sedentary behavior (n = 37, 23.1%), and total energy expenditure (n = 1, 0.6%).

**Table 2:** Study characteristics

Study characteristics	No.	%
**Study designs:**		
Cross-sectional	52	85.2
Longitudinal	9	14.8
**Fields of publication:**		
Ecology	2	3.3
Economics	1	1.6
Public/global health	36	59.0
Medical	12	19.7
Nutrition and physical activity	6	9.8
Psychology	2	3.3
Sustainability	1	1.6
Transportation	1	1.6
**Regions of study locations:**		
Oceania	3	4.9
Europe	27	44.3
North America	13	21.3
Asia	13	21.3
Africa	1	1.6
Multiple	4	6.6
**Study settings:**		
General/not specified	46	75.4
Urban only	9	14.8
Urban and suburban	2	3.3
Urban and rural	4	6.6
**Gender:**		
Both male and female	60	98.4
Female only	1	1.6
**Populations:**		
Children/adolescents/youth (under 18)	8	13.1
Adults (18+)	43	70.5
Older adults (65+)	2	3.3
Children and adults	1	1.6
Not applicable	7	11.5
Specific groups:*		
Adults with specific conditions	10	16.4
Children with specific conditions	1	1.6
Older adults with specific conditions	1	1.6
**Sample sizes:**†:		
<400	18	29.5
400-4999	35	57.4
5000+	8	13.1
**Physical activity outcome measures:**		
Subjective measures	45	73.8
Objective measures	13	21.3
Both subjective and objective measures	3	4.9
**Physical activity outcome domains:**		
Walking and/or biking	13	8.1
Physical activity	92	57.5
Mobility	17	10.6
Sedentary behavior	37	23.1
Total energy expenditure	1	0.6

*Specific groups of population include adults with chronic conditions, with chronic coronary syndromes, of pregnant women, of eating disorder group, and of runners and cyclists; children with congenital heart disease (CHD); older patients with implantable cardioverter-defibrillators (ICD), with type 2 diabetes, subscribers of Leave No Trace (LNT) center for outdoor ethics, a non-profit organization.

†Sample sizse range from 24-4742 000 in the included 61 studies. 18 studies [20-37] with sample sizes lower than 400 and 8 studies [13,38-44] with sample sizes greater than 5000.

### Changes in physical activity amid COVID-19

**Table 3** displays the significant impacts of COVID-19 on walking and biking, physical activity, mobility, sedentary behavior, and total energy expenditure. Most of the reviewed studies showed that the COVID-19 outbreak limited walking and biking, physical activity, and mobility, while increasing sedentary behavior across all age groups worldwide.

**Table 3:** Impact of COVID-19 on physical activity*

			Negative	Positive	Not significant	Not reported
**1**	**Walking and biking**					
	1.01	Walking	[20-23,45-49]		[50]	
	1.02	Walking and biking		[38]*		[40,51]^D^
**2**	**Physical activity**					
	2.01	Light physical activity	[52]*			
	2.02	Moderate physical activity	[20-24,45,46,48,50]	[29]		
	2.03	Vigorous physical activity	[20,22,23,45,46,48,50,52,53]	[29]	[47]	
	2.04	Moderate to vigorous physical activity	[52,53]*			[54]^D^, [55]
	2.05	Meeting the recommended physical activity	47,56]			[57]^D^, [51,58]
	2.06	Meeting the 24-h movement recommendation				[51],[57]*
	2.07	Overall time outdoors				[57]^D^
	2.08	Total physical activity	[20,25-28,46,50,55,56,58-65]	[59]		[35,36,42,66]^D^, [67], [68]^D^
	2.09	Step counts	[21,26,30,31,39,53,69]*			[15]^D^
	2.1	Outdoor physical activity	[70]			[51,57,71]^D^
	2.11	Outdoor play	[70]			[51,57]^D^
	2.12	Physical activity with family		[62]		[57]^D^
	2.13	Exercise/sports	[26]*, [32,49,64,72]			[34,42], [37,73,74]^D^
	2.14	Housework (light, moderate, or heavy)				[34]
	2.15	Labor/physical working (eg, gardening)		[49]		[34]
	2.16	Type of physical activity				[55]
	2.17	Time spent to relax/Leisure activities		[64]		[75]
	2.18	Locations of physical activity				[25], [38]*
	2.19	Outdoor recreation	[76]	[38]*		
**3**	**Mobility**					
	3.01	Overall trips				[20,77]^D^
	3.02	Travel mode/behaviors				[77]^D^
	3.03	Trip purpose				[43,44,77]^D^
	3.04	Use of transportation/Total time spent in transport/Energy expenditure	[48,64]			[77]^D^
	3.05	Bike sharing system usage		[41]		
	3.06	Distance travelled	[76]			[69]*
	3.07	Ridership	[36]			
	3.08	Moving habits				[78]^D^
	3.09	Frequency of outings				[66]
	3.10	Park/green space visitation		[38]*		[79]^D^
**4**	**Sedentary behavior**					
	4.01	Time spent sitting		[20-22,25,29,45,46]	[50]	[54]^D^
	4.02	Screen time (eg, video games, TV watching, computer use)		[22,32,33,48,49,62]	[61]	[35,36,51,54,57,71]^D^
	4.03	Family sedentary behavior				[57]^D^
	4.04	Use of/time spent on social media		[27], [69]*		[51,57]^D^
	4.05	Total sedentary behavior/time		[48,49,58,59]		[66]
	4.06	Non-screen based sedentary activities				[51]^D^
	4.07	Homestay duration		[69]†		
	4.08	E-working time		[48,62]		
	4.09	Resting heart rate	[53]†			
**5**	**Composite**					
	5.01	Total energy expenditure	[20]			

*Subjective Data (no symbol), Objective Data (†), Descriptive Only (D)

Of the 10 studies reporting significant changes in walking and biking during the COVID-19 pandemic, one study that focused on runners and cyclists demonstrated that daily pedestrian (ie, running, walking, and hiking) and cycling recreational activity increased by 291% in Oslo, Norway during the COVID-19 lockdown [38]. The other nine studies indicated significant decreases in walking during confinement [20-23,45-49].

The overall physical activity levels dropped significantly worldwide during COVID-19, including decreases in light [52], moderate and/or vigorous [20-24,45,46,48,50,52], and total physical activity [20,25-28,46,50,55,56,58-65], although one study showed an increase in moderate, vigorous, and total physical activity among university students during confinement [29]. Other studies suggested a significant decrease in the proportion [47] or the number [56] of participants who met the recommended physical activity level (for adults, at least 150 minutes of moderate-intensity aerobic activity according to the Centers for Disease Control and Prevention) during the pandemic, confirming the significant impact of COVID-19 on public health. Further, COVID-19 has resulted in significant decreases in daily step counts [26,30,31,39,52,53,69], outdoor physical activity and outdoor play [70], and exercise/sports [26,32,49,64,72], while several studies indicated significant increases in physical activity with family [62], labor/physical working (eg, gardening) [49], and time spent in leisure activities [64]. Inconsistent impacts of COVID-19 were reported on outdoor recreational activities in two studies. One US study demonstrated a significant decrease in outdoor recreation participation [76], while another European study reported a significant increase in outdoor recreational activity during the pandemic [38].

Studies on mobility showed significant decreases in total time spent in transport [48,64], distances traveled for outdoor recreation [76], subway ridership [40], and the frequency of outings [66]. However, two other studies reported that the COVID-19 outbreak increased the use of the Public Bike Sharing System in South Korea [41] and park and green space visits in Norway [38].

Physical inactivity and sedentary behavior became more prevalent during the COVID-19 pandemic. The reviewed studies showed significant increases in time spent sitting [20-22,25,29,45,46], screen time (eg, video games, TV watching, and computer use) [27,32,33,48,49,62], time spent on social media [27,69], total sedentary time [48,49,58,59], homestay duration [69], and e-working time [48,62]. Another study reported a significant decline in the resting heart rate [53].

Only one study investigated the influence of the COVID-19 pandemic on total energy expenditure. It revealed a significant decrease in energy expenditure among physiotherapy professionals and students during the COVID-19 lockdown [20].

### The roles of community environments

In general, physical activity and trips for all purposes (eg, commuting, shopping, and social/recreational) decreased remarkably since the start of the pandemic. Despite the recognition that supportive community environments are important for promoting active lifestyles, only a small number (n = 11 including three studies with descriptive statistics only, 18%) of the included studies investigated the roles of community environments (**Table 4**) [25,30,34,35,38,42,43,51,55,57,79]. Most of the abovementioned studies were from Europe (n = 5) followed by Canada (n = 3), the US (n = 2), and Asia (n = 1). Although not directly evaluating the environmental effects, 10 additional mobility-related articles (16%) examined the changes in people’s transportation habits (eg, locations they visited) and purposes since COVID-19 [40,41,43,48,64,66,69,76-78]. Four domains of community environments have emerged from these studies to be important for supporting physical activity during the COVID-19 pandemic, including regional locations, dwelling types and density, nature and green spaces, and neighborhood infrastructure (**Table 4**).

**Table 4:** Environmental factors

	Associations with physical activity
Regional locations (urban)	35 (–), 38 (NI), 43 (NI)
Dwelling types and density	30 (living in a flat rather than in a house –; dwelling density NS), 34 (dwelling density NS), 51 (house residence +; dwelling density –), 57 (house residence +)
Nature and green spaces†	25 (–), 51 (–), 55 (+), 66 (+), 79 (+)
Neighborhood infrastructure§	25 (sidewalks and roads, +), 42 (closed infrastructure, no good environments; NI), 51 (proximity to major roads, +)

*Associations with physical activity – Positive (+), Negative (–), Not significant (NS), Not indicated/descriptive (NI).

†Two studies with negative results are for children. and others with positive results are for adults.

§Neighborhood infrastructure excluding nature and green spaces (eg, parks, trails).

Three of the reviewed studies examined regional locations such as urban and rural areas based on inhabitants and ecosystem conditions (ie, vegetation density and canopy covers). One study investigated the relationship between different regional settings (urban, suburban, and rural) and the changes in older adults’ physical activity during the pandemic, which reported a significantly greater decrease in physical activity among those living in urban areas [35]. Another study also reported a rapid decrease in physical activity and the increase in unhealthy behaviors (eg, smoking and alcohol consumption) in both urban (>2000 inhabitants) and rural patient populations with chronic coronary syndromes [38]. However, the decrease in physical activity was greater in urban patients than in rural ones. Descriptively, another US study showed that people in rural areas exhibited less decline in activities at locations such as workplace, transit, retail, and grocery store, but greater decreases in park visits than urban residents [43]. A consistent trend was revealed in a mobility-related article, which found that urban residents with limited access to outdoor recreation/leisure amenities were significantly more impacted by COVID-19 than their rural counterparts [76].

Of the three studies considering dwelling types (detached house vs apartment or flat), two Canadian studies focusing on children and youth populations showed a notable increase in outdoor activities (ie, walking, biking, playing) and overall time spent outdoors among those living in detached houses, compared to residents of apartments [51,57]. The third study conducted in Czech found that heart failure patients living in flats had significant decreases in daily step counts since the onset of the COVID-19, while those living in houses with gardens had less decrease [30]. Additionally, three studies assessed the relationships between residential or dwelling density and changes in physical activity [30,34,51]. Mitra et al. [51] indicated that the outdoor activities of children living in areas with high dwelling density decreased more significantly than those residing in low dwelling density areas. However, the other two studies showed that the changes in physical activity were not associated with residential density [30,34].

Five articles focusing on the association of nature and green spaces with the changes in physical activity during the COVID-19 pandemic showed inconsistent results [25,38,51,55,79]. Two North American studies relating to the child population showed a significant decrease in park visits and outdoor activities in parks or trails [25,51]. The remaining three articles involving adults, or the general population, showed a positive relationship between having/visiting nature or green spaces and physical activity [38,55,79]. Compared to inactive populations, active populations had significantly greater connectedness to nature and nature-relatedness during COVID-19 [55].

In terms of neighborhood infrastructure, three articles considered neighborhood environmental conditions (eg, major roads, sidewalks, and neighborhood amenities) to examine their relationships with the changes in physical activity [25,42,51]. According to Constandt et al. (2020), people in Belgium reported closed infrastructure and the lack of places to exercise as the barriers to exercising since the onset of the pandemic [42]. Dunton et al. (2020) demonstrated that US children had significant increases in physical activity on sidewalks and roads in their neighborhoods, while their physical activity time at parks or trails significantly decreased since the pandemic began [25]. Mitra et al. (2020) showed the proximity to major roads was a barrier to Canadian children, decreasing the odds of participating in outdoor activities. The same study also indicated that access to parks was negatively associated with parent-reported changes in physical activity among children [51].

## DISCUSSION

This rapid review suggested that COVID-19 greatly impacted people’s daily mobility and physical activity patterns. Overall, people worldwide showed decreases in daily physical activities and increases in sedentary time (eg, watching TV, using electronic devices) and time spent at home, compared to the pre-COVID times. As increasing evidence confirms that sedentary behavior, independent of the physical activity levels, accompanies multiple health risks including chronic diseases, obesity, depression, and anxiety [80-83], further efforts are needed to develop effective intervention strategies to break down prolonged sitting time and reduce sedentary lifestyles. On the contrary, specific groups such as undergraduate students and runners/cyclists who used the Strava application showed significant increases in physical activity [29,38]. Housing and neighborhood environments have also been shown to play meaningful roles, with some evidence suggesting their potential to mitigate the negative impact of the pandemic.

### Knowledge gaps recognized from the reviewed studies

Given the time-sensitive nature of published studies on this topic, about a quarter of all the reviewed studies (15 out of 61) relied on descriptive statistics to examine changes in walking, physical activity, sedentary behavior, and mobility behavior, without any statistical tests. Nine out of the 61 (15%) articles included were longitudinal studies comparing the data collected from two different periods before and during COVID-19. Most of the reviewed studies used self-reported or parent-reported survey data asking whether participants’ physical activity had decreased, increased, or remained constant. Seventeen out of the 61 (30%) included studies had limited sample sizes of fewer than 400, ranging from 24 to 315. Even for the articles with larger samples (n ≥400), only 20 studies were representative of the population by using random sampling methods. Lastly, this review identified a limited number of studies (11 out of 61) targeting vulnerable populations such as older adults and children.

While we emphasize the importance of future research on vulnerable populations, the review identified the lack of studies on highlighting the roles of community environments in physical activity and health-related outcomes across different populations in terms of age, gender, income, and health conditions amid COVID-19. For this review, about half (n = 6, 55%) of the eleven environment-related articles focused on specific populations including children (n = 3), older adults (n = 2), and heart failure patients (n = 1). This may highlight the benefits of the community environment for those specific populations who may have limited access to out-of-neighborhood destinations/opportunities. In addition, many existing studies emphasized the importance of neighborhood-level built environments where residents’ daily activities occur (eg, sidewalks and public spaces) on health-related outcomes; and the environmental impacts on those outcomes tend to differ across different populations [84-87]. For example, accessibility to freely available neighborhood resources (eg, walking/biking trails and green spaces) have been shown to impact residents’ behaviors, physical health, and mental health [88,89].

### Built environments and active living during COVID-19

This review identified four environmental factors, including regional locations, dwelling types and density, nature and green spaces, and neighborhood infrastructure related to changes in physical activity. In addition to their community strategies supporting physical activity, the Centers for Disease Control and Prevention provided guidelines on how to be physically active during COVID-19 by visiting parks, pools, or recreation facilities. Although the number of empirical studies on COVID-19 that consider the roles of built environments is small, many leading organizations/initiatives, such as the American Association of Retired Persons (AARP), National Association of City Transportation Officials (NACTO), and professional design groups (eg, MASS, ARUP) have published design guidelines with implementable environmental strategies (eg, curbside seating and increased bike lanes) to cope with the “new normal” following the coronavirus pandemic. These publications address the design conflicts, dilemmas faced, and the potential solutions (eg, tactical urbanism) to provide better outdoor spaces for everyone to stay healthy and engaged, while ensuring safety from infection. For example, NACTO released a resource (ie, Streets for Pandemic Response and Recovery) that includes detailed strategies to redesign and adapt streets for new uses amid COVID-19. AARP published design briefs responding to the impact of COVID-19 on commercial areas, informing local leaders to make their communities more livable and protect their residents from COVID-19 [90]. Their design briefs include Open Streets programs to encourage walking and bicycling to dining and retail areas. A non-profit organization called Bike & Walk Montclair launched a campaign to respond to the pandemic mobility challenges by weighing bike lanes and opening more spaces for pedestrians and bicyclists [91].

### Recommendations for future practice and research

Based on the knowledge gaps mentioned above, longitudinal studies utilizing more vigorous sampling strategies and objective measures of physical activity can help increase the study generalizability and reduce the survey recall bias. More work is needed to investigate the long-term effects of COVID-19 on physical activity and health-related outcomes to further explore how people are recovering from COVID-19. Moreover, further research is needed to investigate the impact of COVID-19 on vulnerable populations (eg, older adults and children) and identify potential social and environmental strategies for promoting active living of everyone after COVID-19.

Given the significance of community environments in maintaining and promoting health behaviors and health outcomes, and ith the limited empirical evidence on the roles of community environments amid COVID-19, more studies are needed to explore how they can help people cope with COVID-19 and how the design dilemma can be addressed in the future. Stronger collaborations between public health and urban planning/design sectors are needed to identify locally implementable environmental strategies that can help respond to the multiple public health challenges that this global pandemic has brought. Drawing from the findings of this review, environmental strategies needing further collaborative efforts include the following.

- Consider potential negative impacts of urbanization and compact developments on physical activity (decreases in physical activity) and health (rapid spread of infections) during pandemics like COVID-19.
- Highlight the importance of providing free and accessible outdoor spaces for all populations (equity).
- Consider shared backyards for residents of high-density dwelling types such as apartments and public/affordable housing, where provision of private outdoor spaces is difficult.
- Consider transforming community vacant land into small parks/green spaces in areas lacking safe outdoor resources to support physical activity and help people cope with mental health problems.
- Consider the importance of safe neighborhood streets and sidewalks to serve as health infrastructure supporting healthy physical activity, especially when other physical activity resources may be unsafe or closed during pandemics like COVID-19.

In addition, many of the pandemic-responsive design/planning strategies proposed and implemented by various organizations and communities have not been examined for their effectiveness. Future research that documents and quantifies the specific benefits and costs of the various pandemic-responsive strategies is needed to develop evidence-based intervention strategies to enable our cities to be better prepared for future pandemics and other public health challenges. In addition to those environmental strategies, educational or promotional programs/events or the use of walking/cycling applications can be encouraged to promote engagement in physical activity.

### Limitations of this rapid review

This rapid review has two major limitations. First, we only used one database for the comprehensive database searches to ensure a timely review. However, Google Scholar searches were also conducted as a supplemental approach to help reduce the possibility of omitting important studies. Second, this review was limited to peer-reviewed quantitative studies in English, which may result in missing relevant qualitative studies and those written in other languages and/or not peer-reviewed. We, however, provided a brief discussion on the grey literature highlighting a few published guidelines most relevant to this review.

## CONCLUSION

Evidence suggests an overall negative impact of COVID-19 on physical activity, with differential effects across different sub-populations. Significant knowledge gaps are also found in the roles of community environments that can promote healthy outdoor activities during the pandemic with reduced safety risks. A major paradigm shift is expected in planning and design to address the dilemma of creating compact and sustainable built environments while providing sufficient protection from infectious diseases and flexibility to accommodate the dynamic needs of outdoor activities during and beyond the pandemic.

## Additional material


Online Supplementary Document
