# Supplementary material for: Impact of COVID-19 on physical activity: A rapid review
Source: J Glob Health. 2022 Apr 30;12:05003. doi: 10.7189/jogh.12.05003 (PMC8979477; doi:10.7189/jogh.12.05003)
Supplement: Online Supplementary Document [file jogh-12-05003-s001.pdf]

Supplementary Table 1. Characteristics of the reviewed studies (n=61)

Ref. Number	1st author, publication year, field of publication	Study design (N/A: not specified or not reported)	Study location (L), setting (S)	Sample size (N), age restriction structure (A), gender (G)	Confounding variables	Environmental factors	Physical activity outcome variables	Statistical analysis	Findings (O: significance, X: insignificance, ∆: counterintuitive N/A: not specified or not reported)	Quality assessment results
77	Beck, 2020, Transport	Cross-sectional	L= Australia; S= General/Not specified	N= 1073; A= 46.3±17.5; G= Both	Demographics (age, children, income); workplace policy; Shopping affected by COVID-19		*Objective and subjective measure* Household trips, use of active modes and motor vehicle	Descriptive statistics; Regression models	N/A	6
54	Meyer, 2020, Public health	Cross-sectional	L= United States; S= General/Not specified	N = 3052; A= 18+; G= Both	Demographics (gender, sex, race), BMI, Smoker, Marital Status, education, employment, chronic conditions, depression, anxiety		*Subjective measure* Physical activity level, sitting time, screen time	Hedges' g effect sizes; confidence intervals; multivariable linear regressions	N/A	4
57	Moore, 2020, Nutrition and physical activity	Cross-sectional	L= Canada; S= General/Not specified	N=1472; A= 5-17; G= Both	Demographics (parental/child and youth); current movement behaviors	Residential type (detached house) significant for outside PA (+), walking and biking (+), outside play (+), inside play (-), overall time outdoor (+), and family physical activity (+)	*Subjective measure* MVPA (days/week) Child movement and play behaviors including outdoor play, outdoor PA/sports, walking and biking (days/week) Screen time (hours/day) Meeting the 24-h movement recommendation	Descriptive statistics; Factorial analysis of variance/t-test; Chi-square; Pearson and point biserial correlation	N/A	5
20	Srivastav, 2020, Global health	Cross-sectional	L=India; S= General/Not specified (sampling from Maharishi Markandeshwar (Deemed to be University))	N= 143	Demographics (age, weight, gender)		*Subjective measure* Vigorous physical activity (MET-min/ week), Moderate physical activity (MET-min/ week), Walking (MET-min/week) Sitting (MET-min/week) Total physical activity with and without sitting (MET-min/week) Energy expenditure (kcal/week)	Wilcoxon signed rank test Measurement: International Physical activity questionnaire-short form (IPAQ-SF); Kolmogorov–Smirnov	Vigorous physical activity (O) Moderate physical activity (O) Walking (O) Sitting (O) Total physical activity with and without sitting (O) Energy expenditure (O)	3
41	Park, 2020, Public health	Cross-sectional	L= Seoul, Korea; S= Urban	N/A	N/A; only ridership data		*Objective measure* Change in Public Bike Sharing System (PBSS) usage per day	Independent t tests; difference-in-differences	Change in PBSS usage per day (O)	4

34	Suzuki, 2020, Public health	Cross-sectional	L= Japan; S= Urban (patients in the hospital in in Kure city, Hiroshima Prefecture)	N = 165; A= 78.6 ± 8.0; G: M=50, F=115	Social demographics; Subjective well-being (Use WHO-5-J); mental component health and health-related quality of life (HRQoL, use SF-12v2) ; Sidewalks; Risk factors associated with decreased PA	Changes in PA since COVID-19 (NS): Residential density (high), access to shops (for good), sidewalks (for presence)	<u>*Subjective measure*</u><br>Changes in PA, SWB, and HRQoL; also mentioned risk factors associated with decreased PA	Two-way repeated-measures ANOVA; paired t-test	NA (only within each group; more active group, equally active group, less active group)	4
55	Lesser, 2020, Environment research and public health	Cross-sectional	L= Canada; S= Urban and suburban	N= 1098; A= over 19 G: M=215, F= 871	Active/Inactive Group; employment status; environment; Location; Childcare; associated barriers; facilitators	Natural relatedness and connectedness to nature (+): significantly greater connectedness to nature (Mdiff = 3.58, SD = 1.42, p = 0.012) and nature relatedness (Mdiff = 0.17, SD = 0.04, p < 0.001) than the inactive population;	<u>*Subjective measure*</u><br>**Active vs Inactive groups**<br>MVPA (min/week)<br>Changes in physical activity since COVID-19 (more/about the same/less)<br>Common types of physical activity<br>Change in type of activity since COVID-19 (very similar/somewhat similar/not so similar)<br>Godin Leisure Score (a leisure score index)	Sample t-tests; one-way ANOVAs	**Active vs Inactive groups**<br>MVPA (NA)<br>Changes in physical activity since COVID-19 (O)<br>Change in type of activity since COVID-19 (very similar/somewhat similar/not so similar) (NA)<br>Godin Leisure Score (a leisure score index) (NA)	5
58	Gallè, 2020, Environment research and public health	Cross-sectional	L= Italy; S= General/Not specified (sampling from Italian universities)	N=1430; A= 22.9 ± 3.5; G: M= 494(34.5%) , F= 936(65.5%)	Socio-demographic features; specific conditions during lockdown; habits of doing housework, going grocery shopping and walking pets; parent's education level; previous PA level; Age, gender, BMI		<u>*Subjective measure*</u><br>Total sedentary time (min/day)<br>Total physical activity (min/week)	The Wilcoxon Signed Ranks Test; Pearson's r value; logistic regression;	Total sedentary time (O)<br>Studying/working (O)<br>Watching TV/DVD (O)<br>Doing leisure activities (O)<br>Using electronic devices (O)<br>Total physical activity (O)	4
75	Fong, 2020, Environment research and public health	Cross-sectional	L= Hong Kong, China; S= Urban	N=590; All age; G: M= 217, F= 373	Demographics (household members, educational level, working status, covering age, gender, average daily studying or		<u>*Subjective measure*</u><br>Change in time spent to relax (e.g. doing leisure activities) - much decreased, decreased, no change, increased, much increased	Chi-square test; multivariate regression models; ANOVA; t-test; Pearson's correlation coefficient; the internal consistency;	Change in time spent to relax (NA)	4

					working hours at home,<br>changes in daily routines)			Cronbach's alpha values of the multi-item scales		
25	Dunton, 2020, Public health	Cross-sectional	L= United States; S= General/Not specified	N=211;<br>A= (5-13) or 8.73±2.58 & older children (9-13);<br>G: M= 100, F= 211	Changes in locations of PA: at home or in the garage (+) and on sidewalks and roads in their neighborhood (+); park or trail (-); yard or driveway (NS), sidewalks and roads outside the neighborhood (NS)		*<u>Subjective measure</u>*<br>Physical activity (much more, somewhat more, about the same, somewhat less, much less);<br>Locations of Physical activity;<br>Sedentary behavior (much more, somewhat more, about the same, somewhat less, much less)	Descriptive statistics;<br>Chi-square test;<br>t-test;<br>linear regression model;<br>Ordinal logistic regression model;<br>Generalized estimating equations (GEE)	Physical activity (O)<br>Locations of Physical activity (at home or in the garage (O), on sidewalks and roads in the neighborhood (O), at park or trail (O), in their yard or driveway (NS), on sidewalks and roads outside the neighborhood (NS)<br>Sedentary behavior (O)	4
51	Mitra, 2020, Health and place	Cross-sectional	L= Canada; S= Urban	N=1472;<br>A: group 1: 5-11 (53%), group 2: 12-17 (47%);<br>G: M=780 (53%), F=692 (47%)	Demographics;<br>Age;<br>Meet MVPA;<br>Sleep duration;<br>Screen time;<br>Household income;<br>parent's employment;<br>Multiple children;<br>gender;<br>dwelling type;<br>dwelling density;<br>access to park;<br>distance to major roads	**Correlates of increased outdoor activities**: house (ref. apartment) (+), dwelling density (-), access to park (NS), distance to major road (+), dwelling density x access to parks (+);<br><br>**Correlates of increased outdoor activities by age group** (children vs. youth): house (+, children), dwelling density (-, both), access to parks (-, children), distance to major road (+, children), dwelling density x access to parks (+, youth)	*<u>Subjective measure</u>*<br>Change in healthy behavior (descriptive; walk or bike, PA or sport outside, PA or sport inside, household chores, playing outside, playing inside, screen time, social media use, other non-screen based sedentary activities)<br><br>**Increased vs decreased outdoor activities**<br>Meet MVPA guidelines<br>Meet 24-h movement guidelines	Cluster analysis;<br>Chi-square test;<br>binomial logistic regression models;<br>multivariate models	Walk or bike (NA)<br>Outdoor physical activity (NA)<br>Outdoor play (NA)<br>Screen time (NA)<br>Social media use (NA)<br>Non-screen based sedentary activities (NA)<br><br>Meet MVPA guidelines (NA)<br>Meet 24-h movement guidelines (NA)	6
29	Romero-Blanco, 2020, Environment research and public health	Cross-sectional;<br>Longitudinal study	L= Spain; S= General/Not specified (sampling from university students in campus residence and	N=213;<br>A=N/A;<br>G: M=41, F=172	Sociodemographic;<br>Anxiety/depression (EuroQol 5D);<br>Mediterranean diet;<br>Living situation;<br>Stage of change		*<u>Subjective measure</u>*<br>Total PA (min/week)<br>Moderate PA (days/week)<br>Moderate PA (min/week)<br>Vigorous PA (days/week)<br>Vigorous PA (min/week)<br>Daily sitting time (minutes)	Bivariate analysis;<br>Kolmogorov–Smirnov test;<br>non-parametric Wilcoxon signed-rank test;<br>Student-Fisher t-test;	Total PA (O)<br>Moderate PA - days (O)<br>Moderate PA - mins (NS)<br>Vigorous PA - days (O)<br>Vigorous PA - mins (NS)<br>Daily sitting time (O)	5

			shared apartments)							
59	Qi, 2020, Environment research and public health	Cross-sectional	L= China; S= Urban and suburban	N=645;<br>A= 31.8±8.6;<br>G: M=250, F=395	Demographic;<br>relevant COVID-19;<br>current PA;	Geographic classification: Suburb vs urban	*<u>Subjective measure</u>*<br>Sedentary time (h/day)<br>Total PA (participation frequency)	Descriptive analysis;<br>ANOVA;<br>Pearson's r;<br>paired t-test	Sedentary time (O)<br>Total PA (O)	5
45	Castañeda-Babarro, 2020, Environment research and public health	Cross-sectional	L= Spain; S= General/Not specified	N=3800;<br>A=18-64；<br>G: M=2054(54%)，F=1746(46%)	Demographic;<br>Subgroup: student, worker, student-worker, did nothing;<br>Subgroup: BMI (underweight, normal, overweight, obese, extremely obese);<br>Subgroup: PA level(vigorous PA, moderate PA)		*<u>Subjective measure</u>*<br>Walking time<br>Moderate PA (categorical; min/week)<br>Vigorous PA (categorical; min/week)<br>Sedentary time (h/day)	T-test;<br>chi-square test	Walking (O)<br>Moderate PA (O)<br>Vigorous PA (O)<br>Sedentary time (O)	4
56	López-Sánchez, 2020, Public health	Cross-sectional	L= Spain; S= General/Not specified	N=163;<br>A=18-64;<br>G: M=47,F=117	Chronic conditions		*<u>Subjective measure</u>*<br>Moderate PA (min/day)<br>Vigorous PA (min/day)	Kołmogorov–Smirnov test;<br>Wilcoxon-signed rank test;<br>Rosenthal's formula	Moderate PA (except age 45-54) (O)<br>Vigorous PA (NS)	2
30	Vetrovsky, 2020, Medical & heart failure	Cross-sectional	L=Czech Republic; S= General/Not specified (patient life circumstances including living in a city with more/less than 10,000 inhabitants, walking distance to countryside less/more than 10min)	N=26;<br>A= 58.8 ± 9.8;<br>G: M=18, F=8	Patient characteristics(age, gender, BMI, health assessments);<br>Patient life circumstances	Daily step count: living in a flat rather than in a house with garden (-) worsened; living in a city more vs less than 10,000 residents (NS), walking distance to countryside less vs more than 10 min (NS)	*<u>Objective measure</u>*<br>Daily step count	Linear mixed-effects models	Daily step count (O)	4
26	Assaloni, 2020, Public Health	Cross-sectional	L= Italy; S= General/Not specified	N= 154;<br>A= 44.8 ± 12.5<br>G: M=84, F=70	Demographics (gender, race, age, marital status, basic anthropometrics (height/weight), BMI, HbA1c), socioeconomic (work, student, sedentary worker, active worker)		*<u>Objective and subjective measure</u>*<br>Godin Scale Score (points)<br>Minutes of exercise (daily)<br>Steps number (daily)	Wilcoxon Signed Ranks test,<br>t-test,<br>Shapiro-Wilk test	Godin Scale Score (O)<br>Minutes of exercise (O)<br>Steps number (O)	4
32	Pietrobelli, 2020, Public health	Longitudinal study	L= Italy; S= General/Not specified	N=41;<br>A=13±3.1	Demographics (age, heigh, weight BMI &		*<u>Subjective measure</u>*<br>Screen time (h/day)<br>Sports (h/week)	Paired t-test;<br>independent two-sample t-tests	Screen time (O)<br>Sports (O)	4

				G: M=22, F=19	waist circumference); Meals, vegetable intake, fruit intake, potato chips, red meat, sugary drinks, screen time, sleep time, sports					
69	Sun, 2020, Public health	Cross-sectional	L= Italy, Spain, Denmark, the UK, and the Netherland; S= General/Not specified	N= 1062;<br>A = N/A;<br>G= N/A	Age, gender, body mass index (BMI), and educational background		*Objective measure*<br>Step counts (daily)<br>Max distance travelled from home (km)<br>Time spent at home (min)<br>Social app use (min)	Kruskal-Wallis tests;<br>post-hoc Dunn's tests;<br>Wilcoxon signed-rank	Step counts (O)<br>Max distance travelled from home (O)<br>Time spent at home (O)<br>Social app use (O)	6
35	Cransac-Miet, 2020, Cardiology	Cross-sectional	L= France; S= Urban and rural	N=195;<br>A=65.5±11.1;<br>G: M=119 (61%), F=76 (39%)	Urban and rural area	Urban vs rural	*Subjective measure*<br>Total physical activity (>% decrease, No change, >25% increase)	Chi square or Fisher exact test;<br>Wilcoxon test;	Total physical activity (descriptive, significance between urban vs. rural)	2
60	Gilic, 2020, Children	Cross-sectional	L= Bosnia & Herzegovina; S= Urban and rural (65% in urban centers and 35% in rural communities)	N= 688;<br>A=15-18;<br>G: M=366<br>F=322	Sociodemographic variables; familial/parental factors		*Subjective measure*<br>Physical activity levels (PAQ-A score)	Kolmogorov–Smirnov test;<br>t-test;<br>Mann Whitney U test;<br>logistic regressions	Physical activity levels (O)	6
27	Elran-Barak, 2020, Environmental research and public health	Cross-sectional	L= Israel; S= General/Not specified	N=315;<br>A=>18;<br>G: M=121, F=178	Demographic (gender, age, marital status); Socioeconomic variables (education, work status, religiosity, num of people in the household); Disease management; Time spent on social media; Medical condition		*Subjective measure*<br>Time spent on social media (h/day)<br>Time spent on online health communities (h/day)<br>Physical activity (times/week) half an hour or more of physical activity	Chi-square test;<br>one-way ANOVA with LSD post-hoc tests;<br>Paired t-test;<br>Ordinal logistic regressions	Time spent on social media (O)<br>Time spent on online health communities (O)<br>Physical activity (O)	3
32	Di Sebastiano, 2020, Psychology	Cross-sectional	L= Canada; S= General/Not specified	N=2388;<br>A=over 18;<br>G: M=234 (9.8%), F=2154 (90.2%)	Demographic information (age, gender, region)		*Objective measure*<br>MVPA (min/week)<br>LPA (min/week)<br>Steps (n/day)	Chi-square test;<br>one-way ANOVA;<br>two-way ANOVAs;<br>partial eta-squared analysis	MVPA (O)<br>LPA (O)<br>Steps (O)	6
52	Hemphill, 2020, Cardiology	Longitudinal study	L= Canada; S= General/Not specified (children from British Columbia Children's Hospital)	N=: 2019 group = 83, 2020 group=82;<br>A= 13± 2.3;<br>G: M=91, F=74	Demographic (age, gender); Diagnosis (coarctation of the aorta, tetralogy of Fallot, transposition of the great arteries,		*Objective measure*<br>Step counts (weekly and daily)	Descriptive statistics;<br>1-way analysis of variance;<br>student's t-test;<br>chi-square test	Step counts (O)	5

					Fontan circulation)					
46	Ammar, 2020, Nutrients, Public health	Cross-sectional	L= Asia, African and Europe; S= General/Not specified	N= 1047; A=18+; G: M=484, F=563	Demographic (age, continent, gender, education, marital status, employment, health state)		*Subjective measure*<br>Vigorous PA (days/week, min/week, MET values)<br>Moderate PA (days/week, min/week, MET values)<br>Walking (days/week, min/week, MET values)<br>Total PA (days/week, min/week, MET values)<br>Sitting (h/day)	Shapiro–Wilks W-test; paired t-test; Cohen's d	Vigorous PA (O)<br>Moderate PA (O)<br>Walking (O)<br>Total PA (O)<br>Sitting (O)	5
66	Gallè, 2020, Environmental research and public health	Cross-sectional	L= Italy; S= General/Not specified (University: The University of Rome La Sapienza, the University of Naples Parthenope and the University of Bari Aldo Moro)	N=2125; A=22.5 ± 0.08; G: M=791, F=1334	Demographic (age, gender); Life Science students and others; education; knowing someone affected by COVID-19		*Subjective measure*<br>PA (decreased, increased, active as before, inactive as before)	Descriptive analysis; student's t-test; chi-square test; logistic regression	PA (NA)	4
39	Pépin, 2020, Medical	Longitudinal study	L= Worldwide (15 countries); S= General/Not specified	N= 742,000; Age and gender vary by country	Age;<br>Gender;<br>Date of Lockdown;<br>Country and province;<br>Baseline and lockdown		*Objective measure*<br>Steps (daily)	Wilcoxon nonparametric test	Steps (O)	6
56	López-Bueno, 2020, Psychology	Cross-sectional	L= Spain; S= General/Not specified	N= 2741; A=34.2±13; G: M=1321(48.2 %) F=1420 (51.8%)	Demographic (age, gender, education, marital status, occupation, body mass);<br>exposure to COVID-19;<br>Along during COVID-19 confinement;<br>Number of previous health risk behaviors;<br>Health risk behaviors during COVID-19 confinement;<br>Week of COVID-19 confinement		*Subjective measure*<br>Screen exposure (more than 2hrs of daily screen time; yes or no)<br>PA (<150 weekly minutes of MVPA; yes or no)	Binomial logistic regression tests; stratified analysis	Screen exposure (NS)<br>PA (O)	4
62	Azizi, 2020, Public health	Cross-sectional	L= Morocco; S= General/Not specified	N=537; A=+18(33.19 ± 12.14);	socio-demographic characteristics (age, marital		*Subjective measure*<br>Daily PA (none, less than 30min, 30min-1h, 1h-1h30min, 1h30min-2)	Mann-Whitney and Kruskal-Wallis test; Stuart-Maxwell test	Daily PA (O)<br>Daily E-working time (O)<br>Daily information tracking time (O)<br>Daily phone calls and SMS (O)	4

				G: M=199; F=338	status, educational status, profession, socio economic level, number of children, presence of disease); before home confinement (484 sample), after home confinement		Daily E-working time (none, less than 2 h, 2-4h, 4-6h, 6-8h, >8h) Daily information tracking time (none, less than 30min, 30min-1h, 1-2h, 2-3h, >3h) Daily phone calls and SMS (none, less than 30min, 30min-1h, 1-2h, 2-3h, >3h) Daily activity time with family members (none, less than 1 h, 1-2h, 2-4h, 4-6h)		Daily activity time with family members (O)	
47	Gallo, 2020, Nutrients	Cross-sectional; Longitudinal study	L= Australia; S= General/Not specified (sampling from the University of Queensland in Brisbane, Australia)	N= 509; A= 19-27; G=Both;	Demographics (gender, age, ethnicity); health practices (former physical activity) ;		*Subjective measure* physical activity level; time of walking or vigorous activity; achieving ‘sufficient’ activity	McNemar’s test; Shapiro–Wilk test	Levels of participation in PA (O); Walking (O); Vigorous PA (N/A); Sufficient PA (O)	5
63	Ruíz-Roso, 2020, Nutrients	Cross-sectional Study; 2020	L= Spain, Italy, Brazil, Chile, Colombia; S= General/Not specified	N= 726; A=10-19; G=Both	Sociodemographic (country, age, sex, household size, maternal education); health practices (former physical activity)		*Subjective measure* Physical activity frequency	Logistic regression	country (o) PA (o)	5
28	Sassone, 2020, Public health	Cross-sectional study	L= Italy; S= General/Not specified	N= 24; A= 62-82; G= Both	Demographics (gender, age)		*Objective measure* Daily physical activity hours	Descriptive analysis	Physical activity (O)	2
48	Zheng, 2020, Public health	Cross-sectional; Longitudinal study	L= Hong Kong, China; S= Urban	N= 631; A= 18-35; G= Both	Demographics (age, height, weight, BMI);		*Subjective measure* Vigorous PA (min/day); Moderate PA (min/day); Walking (min/day); Sedentary behavior (h/day); Sedentary behavior including TV/DVD (h/day), Computer/paperwork (h/day), sitting for transport (h/day)	Descriptive analysis; Wilcoxon Signed-Rank Test;	Vigorous PA (o); Moderate PA (o); Walking (o); Sedentary behavior (o); TV/DVD hours(o); Computer/paperwork hours (o); Sitting for transport hours(o)	6
73	Schnitzer, 2020, Public health	Cross-sectional study	L= Tyrol, Austria; S= General/Not specified	N= 511; A= 25-59; G= Both	Sociodemographic (Age, gender, location, employment)		*Subjective measure* Frequency of sports and exercise	Descriptive analysis	Sports and exercise behavior (NA)	3
40	Park, 2020, Medicine, Public health	Cross-sectional study	L= Seoul, Korea; S= Urban	N/A	Age, purpose		*Objective measure* Ridership change (daily number of passengers)	Unpaired t-test	Ridership change (o)	5

36	Carroll, 2020, Nutrients	Longitudinal study	L= Guelph-Wellington area, Canada; S= General/Not specified	N= 254 (household); A= 18 month+; G= Both	Sociodemographic (ethnicity, household income, marital status)		*Subjective measure* Physical activity, Screen time	Descriptive analysis, Thematic analysis	PA (NA) Screen time (NA)	4
74	Phillipou, 2020, Public health	Cross-sectional study	L= Australia S= General/Not specified	N= 5469; A= 18+; G= Both;	Demographics (age)		*Subjective measure* Exercising behavior	Descriptive statistics; Goodman-Kruskal gamma	Exercising behavior (NA)	3
21	Ruiz-Roso, 2020, Medical, nutrients	Cross-sectional study	L= Madrid, Spain; S= General/Not specified	N= 72; A= 45-77; G= Both	Demographics (gender, age, BMI, Capillary HbA1c)		*Subjective measure* Sitting time (h/week) Walking time (min/week) Moderate PA (min/week)	Correlation test	Sitting time (O) Walking time (O) Moderate PA (O)	4
64	Bourdas, 2020, Public health	Cross-sectional study	L= Greece; S= General/Not specified	N= 5206; A= 18+; G= Both	Demographics (age, weight, height);		*Subjective measure* Daily occupation (min/week); Transportation (min/week); Leisure time activities (min/week); Sporting activities (min/week); Overall PA (min/week)	Paired t–tests; One way ANOVA; Bonferroni pairwise comparisons	Daily occupation (o); Transportation (o); Leisure time activities (o); Sporting activities (o); Overall PA (o);	6
44	Saha, 2020, Public health	Cross-sectional study	L= India; S= General/Not specified	N/A	N/A		*Objective measure* Daily change of different community mobility (percentage): Retail and recreation, Grocery and pharmacy, Parks, Transit stations, Workplaces, Residential	Geo analysis	Daily change of different community mobility (NA)	4
65	Sánchez-Sánchez, 2020， Nutrients	Cross-sectional study	L= Spain S= Urban and rural	N= 1073; A= 16+; G= Both	Demographics (age, BMI, height, weight); Socio-economic (professional status, number of people in the residence, home cooking)		*Subjective measure* Practice of PA (weekly: 1-3 times, 4-5 times, 6+ tunes, do not practice any PA); Time to spend practicing PA in each session (10-30min, 31min-1h, more than 1h, do not practice any PA)	Linear regression	Practice of PA (o) Time to spend practicing PA in each session (o)	4
72	Đogaš, 2020, Medical	Cross-sectional study	L= Croatia, S= General/Not specified	N=3027; A=18+； G= Both	Demographics (age, body weight, height, education); Socio-economic (working status, work rhythm during lockdown, living conditions during lockdown)		*Subjective measure* Frequency of exercise weekly; Duration of exercise (min)	Logistic regression	Frequency of exercise (o); Duration of exercise (o)	4

68	Cancello, 2020, Public health	Cross-sectional study	L= Italy; S= General/Not specified	N=490; A=18+; G= Both	Demographics (gender, age, BMI, educational level, work situation, house size, cohabitation, relatives/himself with COVID-19; habitual physical activity levels food smokers; chronic diseases;		*Subjective measure* PA (unchanged, more than usual, no activity, less than usual), PA change in percentage (increased, decreased, unchanged, sedentary; active subjects before lockdown vs inactive subjects before lockdown)	Descriptive statistics	Physical activity (o)	4
22	Biviá-Roig， 2020， Public health	Cross-sectional study	L=Spain; S=General/Not specified	N=90; A=18+; G= Both	Demographics (Age, height, weight, BMI, week of pregnancy, parity)		*Subjective measure* Changes in PA (days per week, min per day), sedentary activity (hours per day)	Shapiro–Wilk test; nonparametric Wilcoxon tests; Chi-square tests	Vigorous activity (o); Vigorous activity (o); Moderate activity (o); Moderate activity (o); Walking activity (o); Walking activity (o) Sitting (o)	3
42	Constandt, 2020, Public health	Cross-sectional study	L= Belgium; S= General/Not specified	N=13515; A=18+; G= Both	Demographics (Age, gender, highest education, inhouse children); Exercise performance (High active, low active)		*Subjective measure* High vs low active groups Exercise during lockdown, including three categories: i.e., exercising less, exercising as much, exercising more	Descriptive analysis; ordinal logistic regression	High vs low active groups Exercise during lockdown (NA)	5
49	Pisot, 2020, Public health	Cross-sectional study	L=Bosnia and Herzegovina, Croatia, Greece, Kosovo, Italy, Serbia, Slovakia, Slovenia and Spain; S= General/Not specified	N=4108; A=15-82; G= Both	Demographics (age, gender, country)		*Subjective measure* by country: Physical inactivity (h/day) Screen time (h/day), Walking time (h/day), Sports time (h/day), Physical work time (h/day)	Descriptive analysis; Multiple Linear Regression	by country: Physical inactivity (o); Screen time (o); Walking time (o); Sports time (o); Physical work time (Δ)	4
23	Di Stefano, 2020, Medical	Cross-sectional study	L=Sicily, Italy S=General/Not specified	N=268; A=18+, G= Both	Demographics (age; gender; BMI) Levels of PA(MET)		*Subjective measure* Healthy controls and patients with neuromuscular disease MET-vigorous (min/week); MET-moderate (min/week); MET-walking (min/week); MET-total (min/week); MET-MVPA (min/week);	Chi-square test; Mann–Whitney U test; Kruskal–Wallis rank-sum test	MET-vigorous (o); MET-moderate (o); MET-walking (o); MET-total (o); MET-MVPA (o)	3
53	Ong, 2020, Medical	Cross-sectional study	L= Singapore; S= Urban	N= 1824; A= 21–40; G= Both	Sociodemographic (age, gender, ethnicity, family status, household		*Objective measure* Steps (daily counts); MVPA (min/day); Resting heart rate (bpm)	Paired t-test; Pearson's chi-squared test; ANOVA	Steps (o); MVPA (o); Resting heart rate (o)	6

					income in SGD, highest education)					
71	Colley, 2020, Public health	Cross-sectional study	L= Canada; S= General/Not specified	N= 4627; A= 20+; G= Both	Demographics (age, gender)		*Subjective measure* Men vs Women: Exercise outdoors (yes/no); Increased TV use; Increased internet use; Increased video games use; Increased 2-3 types of screens use	Descriptive statistics; Paired t-tests;	Men vs Women Exercise outdoors (NA) Increased TV (NA) Increased internet (NA) Increased video games (NA) Increased 2-3 types of screens (NA)	3
50	Yang, 2020, Public health	Longitudinal study	L= USA; S= General/Not specified	N= 431; A= 18+; G= Both	Demographics (age, gender, BMI, education levels, marital status, income, employment, ethnicity); Covid-19 related symptoms; Tested for Covid-19 (yes/no)		*Subjective measure* PA MET (MET-min/week); Moderate PA (min/day); Vigorous PA (min/day); Active PA (min/day); Walking (min/day); Sedentary time (min/day)	Descriptive statistics; Linear regression analyses	PA MET (o); Moderate PA (o); Vigorous PA (o); Active PA (o); Walking (NA); Sedentary time (NA)	6
37	Ghosh, 2020, Medical	Cross-sectional study	L= India; S= Urban	N=150; A= 18+; G= Both	Demographics (age, gender)		*Subjective measure* Exercise duration (decreased by 25-50%, same as before, increased by 25%, increased by >50%); compared to before lockdown period	Descriptive statistics	Exercise duration (NA)	1
79	Day, 2020, Environmental economics	Cross-sectional study	L= England; S= General/Not specified	N/A	N/A		*Objective measure* Park visitation change (daily %)	Latent class regression analysis	Park visitation change (NA)	5
33	Majumdar, 2020, Medical	Cross-sectional study	L= India; S= General/Not specified	Office Workers N=203 A: 33.1±7.11;G= Both<br><br>Students N=325; A: 22.1±1.66; G= Both	Demographics (age, job experience, sex, marital status, habitual smokers, habitual drinkers)		*Subjective measure* Office workers and students; Cellphone time (h/day); Desktop/laptop time (h/day); TV time (h/day)	Student's t-test	Cellphone (o), Desktop/laptop (o), TV (o)	3
78	Van Nguyen, 2020, Public health	Cross-sectional study	L= Vietnam; S= General/Not specified	N=440; A=N/A G= Both	Sociodemographic (gender, household head's occupation, extra job participation, monthly income, location)		*Subjective measure* Travel plans; Moving habits; Work/study activities;	Descriptive statistics; explanatory factor analysis; Barlett test;	Travel plans (NA); Moving habits (NA); Work/study activities (NA)	3

67	Yamada,<br>2020,<br>Public health	Cross-sectional study	L= Japan;<br>S= General/Not specified	N=500;<br>A=20+;<br>G = Both	Demographics (age, gender)		*Subjective measure*<br>Frequency of outing (%; decrease no change, increase);<br>PA (%; decrease no change, increase);<br>Sedentary behavior (%; decrease no change, increase)	Descriptive statistics	Frequency of outing (NA);<br>PA (NA);<br>Sedentary behavior (NA)	4
43	Sehra,<br>2020,<br>Public health	Cross-sectional study	L= United States;<br>S= General/Not specified	N=22124-83745 (cell phone location data, daily observation);<br>A=N/A;<br>G= N/A	County;<br>days;<br>county factors (number of cases, days since order, rural, population);<br>state factors (age group, black individuals, income, poverty, obesity, without insurance, state GDP, education, inpatient beds per capita, GDP spent on health care, population density)		*Objective measure*<br>Daily cellphone activity compared with baseline over time by category of place (workplace, retail, parks, transit, grocery, residence);	Descriptive statistics;<br>Multivariable linear regression	Daily cellphone activity compared with baseline over time by category of place (NA)	6
13	Tison, 2020,<br>Medical	Cross-sectional study	L= Worldwide;<br>S= General/Not specified	N= 19,144,639 (daily step count)<br>A=N/A;<br>G: N/A	Cities;<br>country;<br>operating system (Android or Apple)		*Objective measure*<br>Mean daily steps (n and % change) by country	Descriptive statistics	Mean daily steps (NA)	3
56	López-Bueno,<br>2020,<br>Sustainability	Cross-sectional study	L= Spain;<br>S= General/Not specified	N = 2042;<br>A = 18+;<br>G= Both	Demographics (age, gender, civil status, occupation, and education)		*Subjective measure*<br>Weekly PA minutes;<br>Adherence to WHO PA guidelines	Mc Nemar Chi-squared tests;<br>independent and paired samples t-test;<br>effect size (Cohen's d) ;<br>receiver operating characteristic (ROC) curve analysis	Weekly PA minutes (o)<br>Adherence to WHO PA guidelines (o)	4
76	Rice, 2020,<br>Urban ecology	Cross-sectional analysis	L= United States;<br>S= Urban and rural	N= 1012;<br>A= N/A;<br>G= Both	Demographics (gender, ethnicity, country of residence, community classification of residence);<br>Behaviors (Average frequency of outdoor recreation, Average distance traveled to		*Subjective measure*<br>**Rural vs Urban cluster vs Urban area residents**<br>Outdoor recreation (frequency, days/week);<br>Distance travelled to participate in outdoor recreation (per session);<br>Distance travelled beyond roads during outdoor recreation (per session)	ANOVA	**Rural vs Urban cluster vs Urban area residents**<br>Outdoor recreation (o);<br>Distance travelled to participate in outdoor recreation (o);<br>Distance travelled beyond roads during outdoor recreation (o);	4

					participate in outdoor recreation, Average distance traveled beyond roads during outdoor recreation, Average outdoor recreation group size)					
70	de Lannoy, 2020, Public Health	Cross-sectional analysis	L= Canada; S= General/Not specified	N=1472; A= 5-17; G= N/A	Province; policies facilitating access to outdoor spaces; policies restricting access to outdoor spaces; number of COVID- 19 cases		<u>*Subjective measure*</u> Time spent outdoors (behavior change score); Outdoor play (behavior change score)	Descriptive statistics	Time spent outdoors (o); Outdoor play (o);	5
38	Venter, 2020, Public Health	Longitudinal Study	L= Oslo, Norway; S= Urban	N= STRAVA runner and cyclist users; A= N/A; G= N/A;	3 years baseline average; Weather; Environmental covariates (Tree cover; vegetation greenness; trail remoteness); Land cover/use type;	Recreational use of greenspaces increase in visits to parks and green spaces for both pedestrians and cyclists (+); Pedestrian increase and tree canopy/greenways (+); Cyclists use trails with greenness (0.2-0.6 NDVI), relative to a baseline preference for less green cover (work-related commutes to recreational purposes);	<u>*Objective measure*</u> Daily pedestrian (run, walk, hike) and cycling (bike) recreational activities recorded >> total activity counts per time interval; Walking and biking activities; Park/green space visits	Descriptive analysis; Linear regression;	Walking and biking (o); Park/green space visits (o); Outdoor recreation (o)	5

Note:

Sallis JF, Johnson M, Calfas KJ et al. Assessing perceived physical environment variables that may influence physical activity. *Res Q Exerc Sport.* 1997; 68:345-351.

Ross, C. E., & Mirowsky, J. (1999). Disorder and decay: The concept and measurement of perceived neighborhood disorder. Urban Affairs Review, 34, 412–432.

Coulton, C. J., Korbin, J. E., & Su, M. (2002). Measuring Neighborhood Context for Young Children in an Urban Area. In T. Revenson, A. D'Augelli, S. French, D. Hughes, D. Livert, E. Seidman, M. Shinn, & H. Yoshikawa (Eds.), Ecological Research to Promote Social Change (pp. 99-126): Springer US.

References:

Ammar, A., Brach, M., Trabelsi, K., Chtourou, H., Boukhris, O., Masmoudi, L., . . . Ahmed, M. (2020). Effects of COVID-19 home confinement on physical activity and eating behaviour Preliminary results of the ECLB-COVID19 international online-survey. *MedRxiv*.

Assaloni, R., Pellino, V. C., Puci, M. V., Ferraro, O. E., Lovecchio, N., Girelli, A., & Vandoni, M. (2020). Coronavirus disease (Covid-19): how does the exercise practice in active people with type 1 diabetes change? A preliminary survey. *diabetes research and clinical practice*, *166*, 108297.

Azizi, A., Achak, D., Aboudi, K., Saad, E., Nejjari, C., Nouira, Y., . . . Marfak, A. (2020). Health-related quality of life and behavior-related lifestyle changes due to the COVID-19 home confinement: Dataset from a Moroccan sample. *Data in brief, 32*, 106239.

Beck, M. J., & Hensher, D. A. (2020). Insights into the impact of COVID-19 on household travel and activities in Australia–The early days under restrictions. *Transport policy, 96*, 76-93.

Biviá-Roig, G., La Rosa, V. L., Gómez-Tébar, M., Serrano-Raya, L., Amer-Cuenca, J. J., Caruso, S., . . . Lisón, J. F. (2020). Analysis of the impact of the confinement resulting from COVID-19 on the lifestyle and psychological wellbeing of Spanish pregnant women: an Internet-based cross-sectional survey. *International journal of environmental research and public health, 17*(16), 5933.

Bourdas, D. I., & Zacharakis, E. D. (2020). Evolution of changes in physical activity over lockdown time: Physical activity datasets of four independent adult sample groups corresponding to each of the last four of the six COVID-19 lockdown weeks in Greece. *Data in brief, 32*, 106301.

Cancello, R., Soranna, D., Zambra, G., Zambon, A., & Invitti, C. (2020). Determinants of the lifestyle changes during COVID-19 pandemic in the residents of Northern Italy. *International journal of environmental research and public health, 17*(17), 6287.

Carroll, N., Sadowski, A., Laila, A., Hruska, V., Nixon, M., Ma, D. W., & Haines, J. (2020). The impact of COVID-19 on health behavior, stress, financial and food security among middle to high income Canadian families with young children. *Nutrients, 12*(8), 2352.

Castañeda-Babarro, A., Arbillaga-Etxarri, A., Gutiérrez-Santamaría, B., & Coca, A. (2020). Physical activity change during COVID-19 confinement. *International journal of environmental research and public health, 17*(18), 6878.

Colley, R. C., Bushnik, T., & Langlois, K. (2020). Exercise and screen time during the COVID-19 pandemic. *Health Rep, 31*(6), 3-11.

Constandt, B., Thibaut, E., De Bosscher, V., Scheerder, J., Ricour, M., & Willem, A. (2020). Exercising in times of lockdown: an analysis of the impact of COVID-19 on levels and patterns of exercise among adults in Belgium. *International journal of environmental research and public health, 17*(11), 4144.

Cransac-Miet, A., Zeller, M., Chagué, F., Faure, A. S., Bichat, F., Danchin, N., . . . Cottin, Y. (2021). Impact of COVID-19 lockdown on lifestyle adherence in stay-at-home patients with chronic coronary syndromes: Towards a time bomb. *International Journal of Cardiology*, *323*, 285-287.

Day, B. H. (2020). The value of greenspace under pandemic lockdown. *Environmental and Resource Economics, 76*(4), 1161-1185.

de Lannoy, L., Rhodes, R. E., Moore, S. A., Faulkner, G., & Tremblay, M. S. (2020). Regional differences in access to the outdoors and outdoor play of Canadian children and youth during the COVID-19 outbreak. *Canadian Journal of Public Health, 111*(6), 988-994.

Di Sebastiano, K. M., Chulak-Bozzer, T., Vanderloo, L. M., & Faulkner, G. (2020). Don't walk so close to me: physical distancing and adult physical activity in Canada. *Frontiers in psychology, 11*, 1895.

Di Stefano, V., Battaglia, G., Giustino, V., Gagliardo, A., D'Aleo, M., Giannini, O., . . . Brighina, F. (2021). Significant reduction of physical activity in patients with neuromuscular disease during COVID-19 pandemic: the long-term consequences of quarantine. *Journal of Neurology, 268*(1), 20-26.

Đogaš, Z., Lušić Kalcina, L., Pavlinac Dodig, I., Demirović, S., Madirazza, K., Valić, M., & Pecotić, R. (2020). The effect of COVID-19 lockdown on lifestyle and mood in Croatian general population: a cross-sectional study. *Croatian medical journal, 61*(4), 309-318.

Dunton, G. F., Do, B., & Wang, S. D. (2020). Early effects of the COVID-19 pandemic on physical activity and sedentary behavior in children living in the US. *BMC Public Health, 20*(1), 1-13.

Elran-Barak, R., & Mozeikov, M. (2020). One month into the reinforcement of social distancing due to the COVID-19 outbreak: subjective health, health behaviors, and loneliness among people with chronic medical conditions. *International journal of environmental research and public health*, *17*(15), 5403.

Fong, B. Y., Wong, M., Law, V. T., Lo, M. F., Ng, T. K., Yee, H. H., . . . Ho, P. W. (2020). Relationships between physical and social behavioural changes and the mental status of homebound residents in Hong Kong during the COVID-19 pandemic. *International journal of environmental research and public health, 17*(18), 6653.

Gallè, F., Sabella, E. A., Da Molin, G., De Giglio, O., Caggiano, G., Di Onofrio, V., . . . Orsi, G. B. (2020). Understanding knowledge and behaviors related to CoViD–19 epidemic in Italian undergraduate students: the EPICO study. *International journal of environmental research and public health, 17*(10), 3481.

Gallè, F., Sabella, E. A., Ferracuti, S., De Giglio, O., Caggiano, G., Protano, C., . . . Liguori, G. (2020). Sedentary behaviors and physical activity of Italian undergraduate students during lockdown at the time of CoViD− 19 pandemic. *International journal of environmental research and public health, 17*(17), 6171.

Gallo, L. A., Gallo, T. F., Young, S. L., Moritz, K. M., & Akison, L. K. (2020). The impact of isolation measures due to COVID-19 on energy intake and physical activity levels in Australian university students. *Nutrients, 12*(6), 1865.

Ghosh, A., Arora, B., Gupta, R., Anoop, S., & Misra, A. (2020). Effects of nationwide lockdown during COVID-19 epidemic on lifestyle and other medical issues of patients with type 2 diabetes in north India. *Diabetes & Metabolic Syndrome: Clinical Research & Reviews, 14*(5), 917-920.

Gilic, B., Ostojic, L., Corluka, M., Volaric, T., & Sekulic, D. (2020). Contextualizing Parental/Familial influence on physical activity in adolescents before and during COVID-19 pandemic: a prospective analysis. *Children, 7*(9), 125.

Hemphill, N. M., Kuan, M. T., & Harris, K. C. (2020). Reduced physical activity during COVID-19 pandemic in children with congenital heart disease. *Canadian Journal of Cardiology, 36*(7), 1130-1134.

Lesser, I. A., & Nienhuis, C. P. (2020). The impact of COVID-19 on physical activity behavior and well-being of Canadians. *International journal of environmental research and public health, 17*(11), 3899.

López-Bueno, R., Calatayud, J., Andersen, L. L., Balsalobre-Fernández, C., Casaña, J., Casajús, J. A., . . . López-Sánchez, G. F. (2020). Immediate impact of the COVID-19 confinement on physical activity levels in Spanish adults. *Sustainability, 12*(14), 5708.

López-Bueno, R., Calatayud, J., Casaña, J., Casajús, J. A., Smith, L., Tully, M. A., . . . López-Sánchez, G. F. (2020). COVID-19 confinement and health risk behaviors in Spain. *Frontiers in psychology, 11*, 1426.

López-Sánchez, G. F., López-Bueno, R., Gil-Salmerón, A., Zauder, R., Skalska, M., Jastrzębska, J., . . . Tully, M. A. (2021). Comparison of physical activity levels in Spanish adults with chronic conditions before and during COVID-19 quarantine. *European journal of public health, 31*(1), 161-166.

Majumdar, P., Biswas, A., & Sahu, S. (2020). COVID-19 pandemic and lockdown: cause of sleep disruption, depression, somatic pain, and increased screen exposure of office workers and students of India. *Chronobiology international, 37*(8), 1191-1200.

Meyer, J., McDowell, C., Lansing, J., Brower, C., Smith, L., Tully, M., & Herring, M. (2020). Changes in physical activity and sedentary behavior in response to COVID-19 and their associations with mental health in 3052 US adults. *International journal of environmental research and public health, 17*(18), 6469.

Mitra, R., Moore, S. A., Gillespie, M., Faulkner, G., Vanderloo, L. M., Chulak-Bozzer, T., . . . Tremblay, M. S. (2020). Healthy movement behaviours in children and youth during the COVID-19 pandemic: Exploring the role of the neighbourhood environment. *Health & place, 65*, 102418.

Moore, S. A., Faulkner, G., Rhodes, R. E., Brussoni, M., Chulak-Bozzer, T., Ferguson, L. J., . . . Vanderloo, L. M. (2020). Impact of the COVID-19 virus outbreak on movement and play behaviours of Canadian children and youth: a national survey. *International Journal of Behavioral Nutrition and Physical Activity, 17*(1), 1-11.

Ong, J. L., Lau, T., Massar, S. A., Chong, Z. T., Ng, B. K., Koek, D., . . . Chee, M. W. (2021). COVID-19-related mobility reduction: heterogenous effects on sleep and physical activity rhythms. *Sleep, 44*(2), zsaa179.

Park, J. (2020). Changes in subway ridership in response to COVID-19 in Seoul, South Korea: Implications for social distancing. *Cureus, 12*(4).

Park, S., Kim, B., & Lee, J. (2020). <? covid19?> Social Distancing and Outdoor Physical Activity During the COVID-19 Outbreak in South Korea: Implications for Physical Distancing Strategies. *Asia Pacific Journal of Public Health, 32*(6-7), 360-362.

Pépin, J. L., Bruno, R. M., Yang, R.-Y., Vercamer, V., Jouhaud, P., Escourrou, P., & Boutouyrie, P. (2020). Wearable activity trackers for monitoring adherence to home confinement during the COVID-19 pandemic worldwide: data aggregation and analysis. *Journal of Medical Internet Research, 22*(6), e19787.

Phillipou, A., Meyer, D., Neill, E., Tan, E. J., Toh, W. L., Van Rheenen, T. E., & Rossell, S. L. (2020). Eating and exercise behaviors in eating disorders and the general population during the COVID-19 pandemic in Australia: Initial results from the COLLATE project. *International Journal of Eating Disorders, 53*(7), 1158-1165.

Pietrobelli, A., Pecoraro, L., Ferruzzi, A., Heo, M., Faith, M., Zoller, T., . . . Heymsfield, S. B. (2020). Effects of COVID-19 lockdown on lifestyle behaviors in children with obesity living in Verona, Italy: a longitudinal study. *Obesity, 28*(8), 1382-1385.

Pišot, S., Milovanović, I., Šimunič, B., Gentile, A., Bosnar, K., Prot, F., . . . Katović, D. (2020). Maintaining everyday life praxis in the time of COVID-19 pandemic measures (ELP-COVID-19 survey). *European journal of public health, 30*(6), 1181-1186.

Qi, M., Li, P., Moyle, W., Weeks, B., & Jones, C. (2020). Physical activity, health-related quality of life, and stress among the Chinese adult population during the COVID-19 pandemic. *International journal of environmental research and public health, 17*(18), 6494.

Rice, W. L., Mateer, T. J., Reigner, N., Newman, P., Lawhon, B., & Taff, B. D. (2020). Changes in recreational behaviors of outdoor enthusiasts during the COVID-19 pandemic: analysis across urban and rural communities. *Journal of Urban Ecology*, *6*(1), juaa020.

Romero-Blanco, C., Rodríguez-Almagro, J., Onieva-Zafra, M. D., Parra-Fernández, M. L., Prado-Laguna, M. D. C., & Hernández-Martínez, A. (2020). Physical activity and sedentary lifestyle in university students: changes during confinement due to the COVID-19 pandemic. *International journal of environmental research and public health, 17*(18), 6567.

Ruíz-Roso, M. B., de Carvalho Padilha, P., Matilla-Escalante, D. C., Brun, P., Ulloa, N., Acevedo-Correa, D., . . . de Oliveira Cardoso, L. (2020). Changes of physical activity and ultra-processed food consumption in adolescents from different countries during Covid-19 pandemic: An observational study. *Nutrients*, *12*(8), 2289.

Ruiz-Roso, M. B., Knott-Torcal, C., Matilla-Escalante, D. C., Garcimartín, A., Sampedro-Nuñez, M. A., Dávalos, A., & Marazuela, M. (2020). COVID-19 lockdown and changes of the dietary pattern and physical activity habits in a cohort of patients with type 2 diabetes mellitus. *Nutrients*, *12*(8), 2327.

Saha, J., Barman, B., & Chouhan, P. (2020). Lockdown for COVID-19 and its impact on community mobility in India: An analysis of the COVID-19 Community Mobility Reports, 2020. *Children and youth services review*, *116*, 105160.

Sánchez-Sánchez, E., Ramírez-Vargas, G., Avellaneda-López, Y., Orellana-Pecino, J. I., García-Marín, E., & Díaz-Jimenez, J. (2020). Eating habits and physical activity of the Spanish population during the COVID-19 pandemic period. *Nutrients*, *12*(9), 2826.

Sassone, B., Mandini, S., Grazzi, G., Mazzoni, G., Myers, J., & Pasanisi, G. (2020). Impact of COVID-19 pandemic on physical activity in patients with implantable cardioverter-defibrillators. *Journal of cardiopulmonary rehabilitation and prevention, 40*(5), 285.

Schnitzer, M., Schöttl, S. E., Kopp, M., & Barth, M. (2020). COVID-19 stay-at-home order in Tyrol, Austria: sports and exercise behaviour in change? *Public Health*, *185*, 218-220.

Sehra, S. T., George, M., Wiebe, D. J., Fundin, S., & Baker, J. F. (2020). Cell phone activity in categories of places and associations with growth in cases of COVID-19 in the US. *JAMA Internal Medicine*, *180*(12), 1614-1620.

Srivastav, A. K., Sharma, N., & Samuel, A. J. (2021). Impact of Coronavirus disease-19 (COVID-19) lockdown on physical activity and energy expenditure among physiotherapy professionals and students using web-based open E-survey sent through WhatsApp, Facebook and Instagram messengers. *Clinical Epidemiology and Global Health, 9*, 78-84.

Sun, S., Folarin, A. A., Ranjan, Y., Rashid, Z., Conde, P., Stewart, C., . . . Simblett, S. (2020). Using smartphones and wearable devices to monitor behavioral changes during COVID-19. *Journal of Medical Internet Research, 22*(9), e19992.

Suzuki, Y., Maeda, N., Hirado, D., Shirakawa, T., & Urabe, Y. (2020). Physical activity changes and its risk factors among community-dwelling Japanese older adults during the COVID-19 epidemic: associations with subjective well-being and health-related quality of life. *International journal of environmental research and public health*, *17*(18), 6591.

Tison, G. H., Avram, R., Kuhar, P., Abreau, S., Marcus, G. M., Pletcher, M. J., & Olgin, J. E. (2020). Worldwide effect of COVID-19 on physical activity: a descriptive study. *Annals of internal medicine*, *173*(9), 767-770.

Van Nguyen, D., Pham, G. H., & Nguyen, D. N. (2020). Impact of the Covid-19 pandemic on perceptions and behaviors of university students in Vietnam. *Data in brief, 31*, 105880.

Venter, Z. S., Barton, D. N., Gundersen, V., Figari, H., & Nowell, M. (2020). Urban nature in a time of crisis: recreational use of green space increases during the COVID-19 outbreak in Oslo, Norway. *Environmental research letters*, *15*(10), 104075.

Vetrovsky, T., Frybova, T., Gant, I., Semerad, M., Cimler, R., Bunc, V., . . . Griva, M. (2020). The detrimental effect of COVID-19 nationwide quarantine on accelerometer-assessed physical activity of heart failure patients. *ESC heart failure, 7*(5), 2093-2097.

Yamada, K., Yamaguchi, S., Sato, K., Fuji, T., & Ohe, T. (2020). The COVID-19 outbreak limits physical activities and increases sedentary behavior: A possible secondary public health crisis for the elderly. *Journal of Orthopaedic Science, 25*(6), 1093.

Yang, Y., & Koenigstorfer, J. (2020). Determinants of physical activity maintenance during the Covid-19 pandemic: a focus on fitness apps. *Translational behavioral medicine*, *10*(4), 835-842.

Zheng, C., Huang, W. Y., Sheridan, S., Sit, C. H.-P., Chen, X.-K., & Wong, S. H.-S. (2020). COVID-19 pandemic brings a sedentary lifestyle in young adults: a cross-sectional and longitudinal study. *International journal of environmental research and public health*, *17*(17), 6035.
